# Postabortion Contraception Acceptance and Associated Factors in Dessie Health Center and Marie Stopes International Clinics, South Wollo Northeast, Amhara Region, 2017

**DOI:** 10.1155/2019/1327351

**Published:** 2019-08-19

**Authors:** Ayele Mamo Abebe, Mesfin Wudu Kassaw, Nathan Estifanos Shewangashaw

**Affiliations:** ^1^Department of Nursing, Debre Birhan Health Sciences College, Debre Birhan 37, Amhara, Ethiopia; ^2^Department of Nursing, College of Health Sciences, Woldia University, Amhara, Ethiopia; ^3^Department of Nursing, College of Health Sciences, Wollo University, Amhara, Ethiopia

## Abstract

**Introduction:**

Abortion is termination of pregnancy before the viability of the pregnancy. It is one of the major causes for maternal mortality in the world and in Ethiopia. Unintended pregnancies which end up in abortion occur due to contraception method nonuse or misuse. To limit unintended pregnancies and avoid repeated abortions promoting immediate postabortion contraception is crucial.

**Objective:**

To assess the proportion of postabortion contraception acceptance among women who got abortion care service and factors associated with it in Marie stopes international clinic and Dessie health center, Dessie, North eastern Amhara, 2017.

**Methods:**

An institutional based cross-sectional study design was conducted from May 1 to May 30, 2017, at Marie stopes international clinics and Dessie health center. A sample of 125 women were selected by means of systematic sampling techniques and 118 abortion clients were interviewed in Marie stopes international clinic and Dessie on the use/acceptance of postabortion family planning (PAFP). Data were collected through pretested structured questionnaire. Data was cleaned and checked. Chi-square test was done to assess the association between dependent and independent variables. Odds ratio was done to assess the strength of association. Frequency tables, pie chart, and graphs were used to present the finding of the study.

**Results:**

From a total of 125 participants recruited, 118 participated in the study while 7 were unwilling to participate in the study, yielding the response rate of 94.4%. Among the 118 study participants, 79 (66.9%) were within the age group 25-34. This study found a strong positive association between Postabortion contraception acceptance and age [P =* 0.007* [X^2^ test=* 9.989,* COR=2.625)]. Study subjects aged 15–24 years were 3 times more likely to accept postabortion family planning as compared with those aged >35 years.

**Conclusion and Recommendation:**

This study revealed that the acceptance of postabortion family planning method was 84%. Age of women, marital status, ever use of history family planning, involvement of others in decision making, and family planning counseling were significantly associated with postabortion family planning acceptance. Therefore it is better to give emphasis on health education about family planning.

## 1. Introduction

### 1.1. Background

Abortion is the ending of pregnancy by removing a fetus or embryo before it can survive outside the uterus. The World Health Organization (WHO) and IFGO recommend that reproductive women who experience abortion should receive contraception assistance, so they can be pregnant in appropriate clinical conditions for the proper development of pregnancy. This requires a minimal interpregnancy interval of six months, mainly to minimize the risk of adverse health outcomes for women and children, such as maternal anemia, premature birth, and low birth weight. These outcomes are widely recognized as being associated with an interpregnancy interval lower than six months [1].

In 2011, the Ministry of Health (MOH) revised the Guidelines for Comprehensive Abortion Care, aiming at standardizing and qualifying women's health care. Aside from the abortion or miscarriage itself, qualified care to women who experience such an episode is considered a priority, primarily to minimize unfavorable outcomes such as maternal and neonatal death, a major challenge that persists in the current health care context [1].

The service includes postabortion care, emergency treatment of incomplete abortion, family planning counseling, STI evaluation and management, HIV testing, and counseling. The major cause for unintended pregnancy which ends up in abortion is nonuse or misuse of contraceptive methods [1].

Although the main aim of availing comprehensive abortion care service in recognized health institutions is to reduce maternal mortality through making abortion safer, the other important point is to introduce, initiate, and link the women with family planning methods after the abortion procedure through postabortion family planning counseling to prevent repeated unplanned pregnancy and abortion because fertility returns within ten days after abortion and the women can get pregnant again [2]. As a result of this, comprehensive postabortion care that includes treatments of incomplete and unsafe abortion, contraceptive, family planning counseling, reproductive and other health services, and abortion related counseling are crucial to prevent unintended pregnancy and repeated abortion

### 1.2. Statements of the Problem

Unsafe abortion is one of the leading causes for maternal mortality worldwide. About 80,000 (13%) maternal deaths per year are thought to be due to abortion complications, one in eight pregnancy-related deaths. An estimated 21.6 million unsafe abortions took place Worldwide in 2012; almost all were done in developing countries [2]. Numbers of unsafe abortions have increased from 19.7 million although the overall unsafe abortion rates remain unchanged at about 14 unsafe abortions per 1000 women aged 15-44 year [2]. This increase in number of unsafe abortions without a corresponding increase in the rate is mainly due to the growing population of women of reproductive age [3].

The national abortion rate is 23 abortions per 1,000 women of reproductive age.This reflects a high demand for abortion related services such as contraceptive methods [1, 4].

According to national family planning guidelines, the total Contraceptive acceptance rate in Ethiopia is 56.2% [2]. Studies done on unsafe abortion in selected health facilities in Ethiopia 2010 showed that fear of side-effects, perceived low risk of conception, inconvenience to use, and sociocultural factors are common reasons for the low use of contraceptives acceptance in general and postabortion family planning in particular [5–7]. However, the status of Postabortion contraception acceptance and factors associated with it in the Amhara region, Dessie health sectors, remains poorly understood.

Therefore, this study is expected to show the status of Postabortion contraception acceptance and associated factors in Dessie city, north eastern Amhara, so as to contribute to evidence based information that can be used by health program managers to design interventions aimed at promoting postabortion family planning among women.

### 1.3. Significance of the Study

Currently, Abortion is becoming a major public health problem in our country. Therefore, this study would be helpful to provide basic information for health care practitioners about Postabortion family planning acceptance. It also helps full for health care provider and other programmers to identify possible factors that affect postabortion family planning acceptance. It might be also helped heath care provider to establish strong postabortion and family planning service linkage based on the finding. It gives baseline information for local official about factors that hindered postabortion family planning acceptance. Additionally it could be contributed to a body of knowledge to further study and other researchers who might conduct a study on related topic and also for organizations working on Abortion and family planning services.

## 2. Literature Review

### 2.1. Introduction

Nearly 22 million unsafe abortions take place every year; these continue to contribute significantly to the global burden of maternal mortality and morbidity [7]. Considering abortion in Africa during 2010-2014, an estimated 8.3 million induced abortion occurred each year in Africa. The annual rate of abortion was estimated at 34 procedures per 1000 women of child bearing age. Abortion rate is roughly 26 for married women and 36 for unmarried women. The annual rate of abortion varies slightly by region, ranging from 38 per 1000 women of child bearing age in Northern Africa to 31 per 1000 in West Africa. In Eastern Middle and Southern Africa, rates are close to the regional average of 34 per 1000. The proportion of pregnancies ending in abortion ranges from 12% in West Africa to 23% and 24% in Northern Southern Africa, respectively. It is 13% and 14% in Middle and Eastern Africa, respectively [8].

### 2.2. Postabortion Contraception Acceptance

The prevalence of Postabortion contraception acceptance is varied. Study conducted in Brazil indicated that of all women enrolled in this study 97.4 percent accepted at least one contraceptive method. Most of them (73.4 percent) had no previous abortion history. From the women who had undergone a previous abortion, 47.5 percent reported undergoing unsafe abortion [2].

Following an intervention to strengthen FP as part of PAC services in rural health districts in Senegal, nearly twice as many PAC clients reported receiving FP counseling after an intervention as before the intervention. In addition, 20% of PAC clients left health care facility with modern contraceptive method compared with the baseline [9].

A nationwide hospital based survey of unsafe abortion, in 9 of the 11 administrative regions of Ethiopia that was conducted from June to December 2010, indicated that the majority of women (87%) were aware of contraceptive methods, but only about half of them ever used a family planning method. Of those pregnancies that ended in abortion, 60% were unplanned and 50% were unwanted. FP Method nonuse was responsible for 78% of pregnancies that occurred. Among those with induced abortion, the most common reason for termination of pregnancy was unmet contraceptive need [2].

Prevalence studies of postabortion contraception done in Ethiopia showed various results. More than 90% of all clients who received an abortion at MSI Clinic in Ethiopia by 2007 left with modern family planning methods [10], while the study done in Tigray from 2007 to 2009 to assess safe abortion care (SAC) monitoring framework showed that only 30% of all women who received abortion services left the facility with a contraceptive method [6].

The study conducted in Burayu, Oromia region, reported that Postabortion contraception acceptance was 88.5%. The usage of contraception is essential in reducing the number of unintended or unwanted pregnancies, which are causes for abortion. Two-thirds of unwanted pregnancies in the developing countries occur among women who are not using any method of contraception [3].

### 2.3. Associated Factor to Postabortion Contraception Acceptance

Despite wide spread adoption of family planning in the developing world, contraceptive use is still very low in sub-Saharan Africa including Ethiopia and other regions. The common cause for induced abortion is unintended or unwanted pregnancy due to varied reasons. Nonuse and misuse of contraception are the major reasons. Contraceptive prevalence rate (CPR) was 63.1%, 25.4%, and 14.7% worldwide, in Africa and in Ethiopia, respectively. This indicates that there was low utilization of contraception [11].

Studies revealed that various factors influence acceptance of postabortion family planning method. Age of women, educational status of women, occupational status, and marital status are among sociodemographic factors as identified by many studies. Family planning counseling by health care provider, previous history of abortion, and previous history use family planning are also indicated as reproductive abortion and related factors [2, 10, 12].

The study conducted in Pakistan revealed that majority of the PAC seekers aged between 25 and 34 years had 1 to 4 children (less educated and housewives). On the other hand, a study, done in Turkey, showed that postabortion contraception was influenced by age groups, educational levels, parity, future fertility plan, and previous induced abortion [4, 13].

Studies in Ethiopia indicated that women seeking induced abortion had a mean age of 23, and the majorities (57%) were single among women seeking induced abortion; only 24% of them reported contraceptive use before the current pregnancy. Furthermore, health care services factors play roles in promoting increased use of postabortion contraception. A cross-sectional survey, in two regions of Ethiopia (2002/3), showed that 53.4% of clients left health care facilities counseled about family planning and 44% with contraceptives [14].

From the study done to assess the future potential capacity and quality of PAC service delivery in public health facilities in three regions of Ethiopia, 23 percent health facilities reported they provide postabortion contraceptive service regularly. The rest of the facilities either rarely or never provide contraceptives. Postabortion counseling was reported to be a regular service provided by three fourths of the facilities but many of the health staffs (46%) who provide contraceptive method or counseling do not have special training in contraceptive counseling or provision [3].

From the study made in Addis Ababa on clients presented for abortion related services, only 57% were using contraceptive before presenting for abortion services. Among them, short-term method of contraception was common and almost one third reported one or more previous abortions. Women seeking safe termination are relatively young [8].

The study conducted in Burayu concluded that most of the women who seek abortion care service in the health centers were very young women. About 209 (53.5%) couples decide on Family planning together, whereas 165 (42.2%) women reported to decide by themselves. Most of women got counseling services more likely accepting PAFP than those who did not get counseling. Counseling, decision on family planning, abortion methods, and income of respondents were found to be determinant factors [15].

## 3. Objective

### 3.1. General Objective

The overall objective of the study is to assess the proportion of postabortion contraception acceptance among abortion care service clients and factors associated with it in Marie stopes international and Dessie health center, Dessie, Amhara, 2017.

### 3.2. Specific objectives

1. The first is to determine the proportion of postabortion contraception acceptance in Marie stopes international and Dessie health center, Dessie, Amhara, 2017.
2. The second is to identify factors associated with postabortion contraception acceptance in Dessie health in Marie stopes international and Dessie health center, Dessie, Amhara, 2017.

## 4. Methods

### 4.1. Study Area

The study was conducted in Marie stopes international clinics and Dessie health center, which provide postabortion service in Dessie Town. It is found at north direction of Amhara region about 480 km from Bahir Dar and 401 km from Addis Ababa, Capital City of Ethiopia. In Dessie town there are 2 general government hospitals, 3 private hospitals, 5 governmental health centers, and more than 4 private clinics. The health center has MVA service and medical abortion for early trimester abortions after which patients are counseled about different options of contraceptives and allowed to choose and use them.

### 4.2. Study Design and Period

Institutional based cross-sectional study was conducted from May 1 to May 30, 2017.

### 4.3. Source Population

All reproductive age women (15-49) got postabortion care service in Marie stopes international clinics and Dessie health centers.

### 4.4. Study Population

The study population was all reproductive aged women who received abortion care service in Marie stopes international clinics and Dessie health centers during the study period.

### 4.5. Eligibility Criteria

#### 4.5.1. Inclusion Criteria

These included women who attended abortion service.

#### 4.5.2. Exclusion Criteria

These included those who were physically or mentally unable to make interview.

### 4.6. Sample Size and Sampling Techniques

#### 4.6.1. Sample Size Determination

Sample size is calculated by using single population proportion formulas. Sample size was determined by taking p-value30% from related literature of postabortion contraceptive acceptance services in Tigray [16]. Confidence interval (95%) or Z=1.96 value of this formula N-sample size

(1) Margin  of  error=5%n=za22p1−pd2n=1.9620.31−0.30.052n=323

The final sample size will be made by correction formula nf=n/1+ (n/N)

(2) $$\begin{array}{c} nf=\frac{323}{1}+\frac{323}{176}=114 \end{array}$$

Adding 10% nonresponses

(3) $$\begin{array}{c} nf=125 \end{array}$$

#### 4.6.2. Sampling Techniques

Sampling method was random, probability sampling because the study subjects are rare (abortion clients are rare) at the time of the data collection. All consecutive patients seeking postabortion care at sampling units at randomly selected days (i.e., Monday, Tuesday, Friday, and Saturday) during exit from abortion care service unit were included in the study. The study subjects were clients who received abortion care from Maria stopes international clinics and Dessie health centers from 1st of May till 30th of May, 2017. The total sample size was allocating to each health facilities based on the proportion of average clients served, allocated based on the previous three-month average clients' load.

### 4.7. Variables of the Study

#### 4.7.1. Dependent Variable

This was postabortion contraceptive acceptance.

#### 4.7.2. Independent Variable

*Sociodemographic Characteristics*. These were age, religion, marital status occupation, educational status, Residence, and Ethnicity.

*Reproductive Health Related Variables*. These were parity, history of family planning use, previous history of abortion, counseling by health care provider, decision making process, and reasons for not using family planning.

### 4.8. Operational Definition

*Acceptance of FP*. This is the use of contraceptive method among clients for abortion care in the health care facility.

*Abortion*. This is the termination of pregnancy before the viabilities of the fetus.

*Postabortion Contraception*. This is the use of contraceptives immediately after any abortion care procedure.

### 4.9. Method of Data Collection

Interviewer-administered structured questionnaires questions were developed based on review of literature. The questionnaire was prepared in English and then translated into Amharic keeping the content of the question using understandable words, as the study participants speak Amharic language. Before conducting the main study, pretest was carried out in Hayek health center which is not included in the main study. Pretest was done on 5% of the sample size before the data collection period. Based on the pretest result, the questionnaire was modified as necessary. The interview was filled by asking the women after they got the abortion care service. The filled interview was checked for their completeness and consistency (Table 1).

### 4.10. Data Entry and Analysis

Regarding descriptive statics means, proportion was done to see the distribution of sociodemographic characteristics. Chi-square was done to assess the association between postabortion family acceptance and independent variables. Odds ratio was done to assess the strength of association. Frequency tables, graphs, and pie charts were used to present the finding of the study.

### 4.11. Data Quality Assurance

We used pretested and structured questioners. The questionnaire was pretested among 5% of the sample size population in Haik. Investigators collected the data. Completed questionnaires were checked on a daily basis by investigators.

### 4.12. Ethical Considerations

Ethical clearance letter was obtained from Alkan Health Science, Business, and Technology College. A formal letter was given to Mario stopes international and Dessie health center. Informed consent was obtained from study participants before the interview. The participants of the study were told that they can refuse to continue or escape questions whenever they want, and confidentiality of the information they give was kept.

### 4.13. Dissemination of Result

The results of this study will be communicated to Zonal health department and other concerned bodies through reports. The study will be presented to Alkan Health, Business, and Technology College members

## 5. Result

### 5.1. Sociodemographic Characteristics

From a total of 125 participants recruited, 118 participated in the study while 7 were unwilling to participate in the study, yielding the response rate of 94.4%. Among the 118 study participants, 79 (66.9%) were within the age group 25-34. Half of the respondents, 63 (53.4%) were followers of Orthodox, 45 (38.1%) were Muslim, and 10 (8.5%) were protestants. The predominant ethnic group was Amhara 76 (64.4%). Majority of the respondents 69 (58.5%) were single; 73 (61.9%) of the study participants were unemployed (see Table 2).

### 5.2. Reproductive Health, Family Planning, and Abortion Related Characteristics of Participants

Among the study participants, 44 (37.3%) of them had not a history giving birth and 109 (92.4%) of this study participants reported that they had no history of previous abortion (Table 3).

### 5.3. Reason for Not Using Family Planning

**Figure 1:**
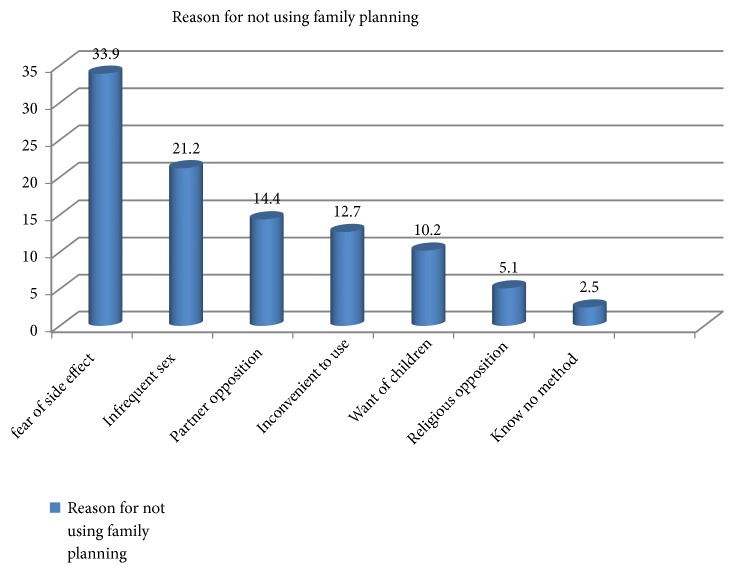
Study participants reason of not using family planning method among abortion service clients in Dessie, Amhara regional state, Ethiopia, 2017.

33.9% of this study participants reported that they were not using family planning method because of fear of its side effect (Figure 1).

### 5.4. Respondents Age and Acceptance Family Planning

**Figure 2:**
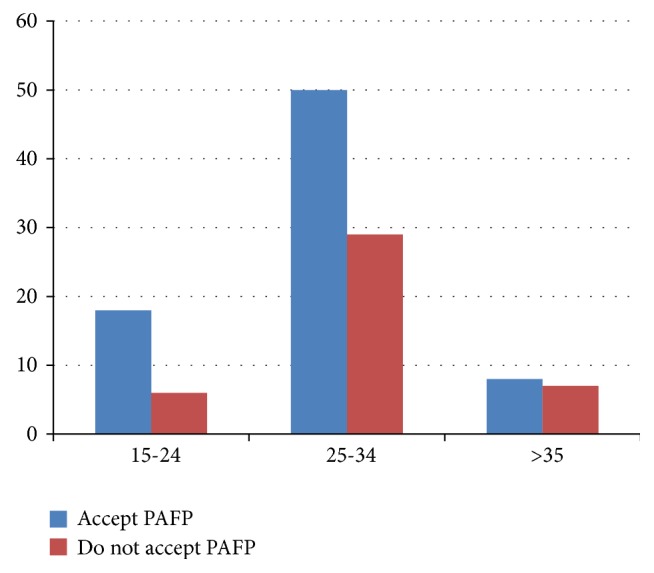
Age category and acceptance among postabortion service clients in Dessie, Amhara, Ethiopia, 2017.

Among the 118 study participants, 79 (66.9%) were within the age group 25-34 which group had high acceptance of post abortion family planning (see Figure 2).

### 5.5. Acceptance of Postabortion Contraception

**Figure 3:**
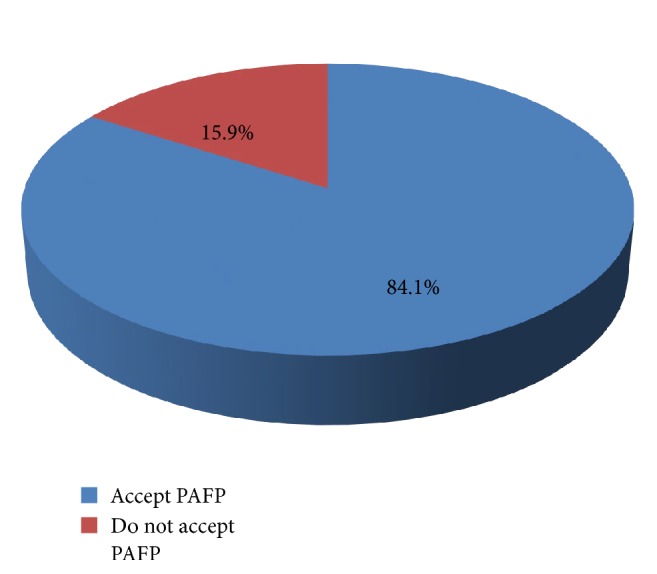
Acceptance of postabortion family planning among abortion service clients in Dessie, Amhara, Ethiopia, 2017.

The overall acceptance of Postabortion contraception acceptance among abortion service clients in Marie stopes international and Dessie health center was 84.1 % (see Figure 3).

### 5.6. Factors Associated with Acceptance of Postabortion Contraceptive

This study found a strong positive association between Postabortion contraception acceptance and age [P =* 0.007* [X^2^ test=* 9.989,* COR=2.625)]. Study subjects aged 15–24 years were 3 times more likely to accept postabortion family planning as compared with those aged >35 years and the odds ratios of accepting postabortion family planning among aborted women in the age group of 25-34 were 2 times higher than those aged >30 years.

Females who decide on family planning had significant association with Postabortion family planning acceptance. Females who made independent decision were two times more likely to accept PAFP compared with those decided by husband or both of them [P =* 0.022*[X^2^ test=* 7.679,* COR=1.695)] (see Table 4).

## 6. Discussion

This study revealed that the magnitude of acceptance of postabortion contraception was 84%. Similar finding was reported in many studies [5, 15, 17]. On the other hand, the current study finding was higher than the finding of studies done in Pakistan (72.9%)[4] and lower than the studies done in Brazil (97.4%)[18]. This variation of findings across various studies might be due to cross-cultural limitations of diagnostic tools and reporting biases, differences in socioeconomic environments. Prevalence estimates also are likely to be influenced by stigma and discrimination.

Compared to general population, it can be assumed that acceptance of family planning is higher among clients who visit health facility for abortion, because of their condition. Comparing this study results with a Community based findings, we found our rates to be higher across the board: EDHS 2000 (84.1% vs. 5.76%), EDHS 2005 (84.1% vs. 15.9%), EDHS 2011 (84.1% vs. 35.6%), and EDHS 2014 (84.1 % vs. 39.1%) [19]. Several factors can explain much higher acceptance of family planning among women who faced abortion. First, most of the women who came to get abortion care service had unintended/unplanned pregnancy; they all need to use contraception to avoid similar incidents. Second majority (80%) of these study participants were within the group 15-34; as a result they might not have stable relationships which make them at risk for unintended pregnancy which is unacceptable by the society.

This study found a strong positive association between Postabortion contraception acceptance and age [P = 0.007 [X^2^ test= 9.989, COR=2.625)]. Study subjects aged 15–24 years were 3 times more likely to accept postabortion family planning as compared with those aged >35 years and the odds ratios of accepting postabortion family planning among aborted women in the age group of 25-34 were 2 times higher than those aged >30 years. Similar relations were reported from the studies conducted in Ethiopia, Nepal, Vietnam, and Brazil [5, 15, 18, 20]. The possible reasons may be that young women have a greater probability of having unintended pregnancy which will end up with abortion.

In agreement with studies reported from 13 developing countries and Ethiopia in this study single women had high acceptance rate of postabortion family planning method [8, 15]. Participants who were single were 2 times more likely to accept postabortion family planning as compared with participants who were married [P = 0.0034 [X^2^ test= 4.494, COR=2.398)]. The finding implies that females with unstable marital relationship increase acceptance of postabortion family planning. This may be due to first unmarried women may not use contraceptive as most of them have casual sex. Second those who are married are more likely to have settled and share the burden of increasing living cost which deceased fear of pregnancy and delivery; on the other hand single women have fear related with pregnancy because they do not have stable relationship and pregnancy without marital relationship is culturally unacceptable and discouraged.

This study result showed that females who decide on family planning had significant association with Postabortion family planning acceptance. Females who made independent decision were two times more likely to accept PAFP compared with those decided by husband or both of them [P =* 0.022 *[X^2^ test=* 7.679,* COR=1.695)]. This study finding is supported by studies done in Addis Ababa and Burayu [15, 21]. In addition, a study done in three regional states of Ethiopia, 2010, depicted that women make independent decision 46.5% regarding seeking postabortion care higher in magnitude than partner involvement decision making 42.4% [10]. The observed higher acceptance of postabortion family planning among females who passed independent decision might be explained as follows: since self-decision provides free space of choices females may tend to accept contraceptive.

Consistent with the study result in Burayu town which indicated that there is no significant association between participants family planning use history and acceptance of postabortion family planning, this study result showed that there is strong positive association between Postabortion contraception acceptance and family planning use history [15]. In this study females who had a use family planning had high acceptance postabortion family planning method when compared to those females who had not a history of family planning use [P= 0.000 [X^2^ test= 63.621, COR=53.04)]. This might be due to the fact that those females who had a history of use family planning have better awareness on the method and side effect of family planning which facilitate choice of family planning, as supported by the result of this study which revealed that majority (56%) of this study participants reports fear of side effect which is the reason for why they did not accept postabortion family planning.

This study further depicts that women who did not get counseling were 4 [P =* 0.03*[X^2^ test=* 4.333,* COR=2.369)] times less likely to accept postabortion family planning method compared with women who had counseled. This finding was supported by studies done in Ethiopia, Nepal, Brazil, and thirty countries [2, 5, 8, 15, 17, 22, 23]. This implies that family planning counseling is critical to raise acceptance of postabortion family planning method.

In this study occupational status, educational status, residence, place of abortion, those who ever give birth, and those who ever had abortion had no statistically significant association with acceptance postabortion family planning method as other studies found out [8, 22, 24, 25].

## 7. Strength and Limitation

### 7.1. Strength

- The first point is use of adopted, validated, and translated questionnaire.
- The second point is the limited study in the area.

### 7.2. Limitations

- The first point is social desirability bias.
- The second point is cross-sectional study design that established causal relationship impossible.

## 8. Conclusion and Recommendation

### 8.1. Conclusion

This study revealed that the acceptance of postabortion family planning method was 84%. Age of women, marital status, ever use of history family planning, involvement of others in decision making, and family planning counseling were significantly associated with postabortion family planning acceptance. Occupational status, educational status, residence, place of abortion, ever giving birth, and history of abortion had no statistically significant association with acceptance postabortion family planning method.

### 8.2. Recommendation

Based on this study finding the following recommendation was given.

*(1) To Local Officials*

- It is better to strengthen support health facility in establishing relation with school and community workers.

*(2) Marie Stopes International Clinic and Dessie Health Center*

- It is better to address young females through establishing link with schools.
- It is better to strengthen family planning counseling services.
- It is better to support females in decision making skills and process through establishing strong linkage with health extension and community workers.

*(3) Health Educator and Researcher*

- It is better for health educators to provide continuous health information about postabortion family planning methods and their side effects.
- Researchers should further investigate other variable links with postabortion family planning and repeat the study with a comparison or control group to see the difference and the direction of relation.

## Figures and Tables

**Table 1:** 

Health facility	Study participants	%
Marie stopes international clinic	101	80.8%
Dessie health center	24	19.2%

**Table 2:** Distribution of sociodemographic characteristics among abortion care service clients in Marie stopes international and Dessie health center, Dessie, North eastern Ethiopia, 2017 (n=118).

Variables	Frequency (n=118)	%
*Age*		
18-24	24	20.4
25-34	79	66.9
>35	15	12.7
*Ethnicity*		
Amara	76	64.4
Tigray	13	11.0
Oromo	29	24.6
*Religion*		
Orthodox	63	53.4
Muslim	45	38.1
Protestant	10	8.5
*Residence*		
Dessie	83	70.3
Out of Dessie	35	29.7
*Marital status*		
Single	69	58.5
Married	49	41.5
*Education*		
Literate	91	77.1
Illiterate	27	22.9
*Occupational status*		
Employed	45	38.1
Unemployed	73	61.9

**Table 3:** Reproductive health, family planning, and abortion related characteristics of participants, Dessie, Amhara, Ethiopia, 2017.

Variables	Frequency (n=118)	%

*Have you ever given birth?*		
Yes	44	37.3
No	74	62.7
*Previous history of abortion*		
Yes	9	7.6
No	109	92.4
*Who give decision about use of FP∗?*		
Wife	61	51.7
Husband	36	30.5
Both	21	17.8
*Have you ever been counseled by HP health about FP?*		
Yes	70	59.3
No	48	40.7
*Have you ever used FP?*		
Yes	62	52.5
No	56	47.5

*FP∗:* family planning.

**Table 4:** Factors associated with Postabortion contraception acceptance among abortion service clients in Dessie, Amhara, Ethiopia, 2017.

Variables	Postabortion contraception acceptance		OR		
	Yes	No	COR	X^2^ test	P-value

*Age*					
15-24	18	6	2.625	*9.989*	*0.007∗∗*
25-34	50	29	1.97		
35	8	7	1		
*Education*					
Literate	47	44	1.335	0.192	0.661
Illiterate	12	15	1		
*Marital status*					
Single	43	26	2.398	*4.494*	*0.034∗*
Married	20	29	1		
*Occupational status*					
Employed	24	21	1.385	.0.447	0.503
Unemployed	33	40	1		
*Residence*					
Dessie	44	39	1.065	0.002	0.964
Out of Dessie	18	17	1		
*Place of abortion*					
Marie stopes International	82	13	1.752	0.410	0.521
Dessie health centre	18	5			
*Have you ever given birth?*					
No	42	32	1.727	1.528	0.216
Yes	19	25	1		
*Have you ever been counseled by HP about FP? *					
Yes	44	26	2.369	*4.333*	*0.03∗*
No	20	28	1		
*Have you ever used FP?*					
Yes	52	10	53.040	*63.621*	*0.000*∗∗∗
No	5	51	1		
*Who give decision about use of FP*					
Wife	37	24	1.695	*7.679*	*0.022∗*
Husband	22	14	1.728		
Both	10	11	1		

∗∗∗*0.000*  ∗∗<*0.02*  ∗<*0.05*.

## Data Availability

Data supporting the conclusions of this article are available by request to Ayele Mamo. The relevant raw data will be made available to researchers wishing to use them for noncommercial purposes.

## Acronyms

DMPA: Depot-medroxyprogesterone acetate
FIGO: Federation of international gynecology and obstetrics
FP: Family planning
IUD: Intrauterine device
LARC: Long acting reversible contraceptives
MMR: Maternal mortality rate
MOH: Ministry of Health
MSIE: Marie stopes international clinic in Ethiopia
MVA: Manual vacuum aspiration
OR: Odd ratio
PAFP: Postabortion family planning.

## Supplementary Materials

Supplementary Materials Supplementary Material in the research included consent form, questionnaire in English version, and questionnaire in the Amharic version (local language).

## References

[1] Van Look P. F. A., Cottingham J. (2013). The World Health Organization’s safe abortion guidance document. *American Journal of Public Health*.

[2] Kesetebirhan A.

[3] Seid A., Gebremariam A., Abera M. (2013). Integration of family planning services within post abortion care at health facilities in dessie –North East Ethiopia. *Science, Technology and Arts Research Journal*.

[4] Khurram S.

[5] Puri M.

[6] Central Stastical Agency, Ethiopia Demographic Health Servey, 2011

[7] WHO

[8] Singh S. (2006). Hospital admissions resulting from unsafe abortion: estimates from 13 developing countries. *The Lancet*.

[9] Curtis S. L., Evens E., Sambisa W. (2011). Contraceptive discontinuation and unintended pregnancy: an imperfect relationship. *International Perspectives on Sexual & Reproductive Health*.

[10] Melkamu Y., Betre M., Tesfaye S. (2010). Utilization of post-abortion care services in three regional states of Ethiopia. *Ethiopian Journal of Health Development*.

[11] WHO Regional Office for Africa, I.o.O.B.f.P.o.M.-T.C.T.o.H., 2017

[12] HIV/AIDS, J.U.N.P.o., AIDS scorecards: overview: UNAIDS report on the global AIDS epidemic 2010, UNAIDS, 2009

[13] Post abortion family planning Tureky 2012

[14] Federal Democratic Republic of Ethiopia

[15] Andarge F.

[16] Monitering safe abortion care service provision in Tigray, Ethiopia, 2009

[17] Worku S., Fantahun M. (2007). Unintended pregnancy and induced abortion in a town with accessible family planning services: The case of Harar in eastern Ethiopia. *Ethiopian Journal of Health Development*.

[18] Carneiro Gomes Ferreira A. L., Impieri Souza A., Evangelista Pessoa R., Braga C. (2011). The effectiveness of contraceptive counseling for women in the postabortion period: an intervention study. *Contraception*.

[19] Worku A. G., Tessema G. A., Zeleke A. A., Räisänen S. H. (2015). Trends of modern contraceptive use among young married women based on the 2000, 2005, and 2011 ethiopian demographic and health surveys: a multivariate decomposition analysis. *PLoS ONE*.

[20] Vu L. T., Oh J., Bui Q. T., Le A. T. (2016). Use of modern contraceptives among married women in Vietnam: a multilevel analysis using the multiple indicator cluster survey (2011) and the Vietnam population and housing census (2009). *Global Health Action*.

[21] Prata N., Holston M., Fraser A., Melkamu Y. (2013). Contraceptive use among women seeking repeat abortion in Addis Ababa, Ethiopia. *African Journal of Reproductive Health*.

[22] Tarekegn S. M., Lieberman L. S., Giedraitis V. (2014). Determinants of maternal health service utilization in Ethiopia: analysis of the 2011 Ethiopian Demographic and Health Survey. *BMC Pregnancy and Childbirth*.

[23] Benson J., Andersen K., Brahmi D. (2017). What contraception do women use after abortion? An analysis of 319,385 cases from eight countries. *Global Public Health*.

[24] Sundaram A., Vlassoff M., Bankole A., Remez L., Gebrehiwot Y. (2010). Benefits of meeting the contraceptive needs of Ethiopian women.. *Issues in brief (Alan Guttmacher Institute)*.

[25] Singh S.

