# Supplementary material for: Postabortion Contraception Acceptance and Associated Factors in Dessie Health Center and Marie Stopes International Clinics, South Wollo Northeast, Amhara Region, 2017
Source: Int J Reprod Med. 2019 Aug 19;2019:1327351. doi: 10.1155/2019/1327351 (PMC6719265; doi:10.1155/2019/1327351)
Supplement: Supplementary Materials — Supplementary Material in the research included consent form, questionnaire in English version, and questionnaire in the Amharic version (local language). [file 1327351.f1.pdf]

# Supplementary Materials

## Consent form

Assessments of post abortion contraception acceptance and associated factors

**I: Individual consent form**

Section 0: Questionnaire Identification

001-Date___/____/______

002Qustionnaire Identification Number ____________

003 health institution __________

Introduction

My name is ________________________. I am student in Alkan health science and Business College, public health department. I am interviewing abortion care service clients here about acceptance of post abortion family planning and associated factors for research purpose. This study is designed to generate information for family planning in the area and elsewhere with similar characteristics. To attain this purpose, your honest and genuine participation by responding to the question prepared is very important highly appreciated. Your answers are completely confidential. Your name will not be written on this form. You can refuses to answer a single question, more than one question to the extent to stop the interview at any step if you are not comfortable. However your honest answer to these Questions will help me to understand the PAFP acceptance and associated factors .The interview will take about 15- 25 minutes.

Are you willing to participate?

IF the answer is yes _ Continue

No _ Stop

Name of health institution___________________________

Signature of the interviewee certified that respondent has given informed consent verbally

Checked by name________________ sig_________________date________________

## Supplementary Materials 1: English version Questionnaires

We are the graduate student from Alkan health science collage of public health and collect information on the post abortion contraception acceptance and associated factors in Dessie health center. We should be grateful if you would complete this questionnaire being as honest as possible.

Socio demographic data

1. Age : A. 15-19 B. 20-24 C. 25-29 D. >30
2. Ethnicity :

   A. Oromo B. Tigris

   C. Amhara

3. Religion
   A. Orthodox Christian
   B. Protestant Christian
   C. Catholic
   D. Muslim
   E. Others ______________

4. Where is your current residence?

   A. Dessie

   B. Region

   C. Rural area

5. What is your current marital status?

   A -single C -separated/divorced

   B -married D- widowed

6-what is your level of education?

A. Cannot read and write
B. Able to read and write/informal education
C. Primary school(1-8 grade)
D. High school (9-12 grade)
E. collage /University graduate

7-what is your current occupation?

A. House wife
B. Government employee
C. Student
D. Daily laborer
E. Others (specify)

**Part 2, RH data**

8. Have you ever given birth?

A. Yes
B. No

9. Have you ever had an abortion?

A. Yes

B. No

**Part 3. Family planning related data**

10-Have you ever heard about any family planning methods?

A. Yes

B. No

11-Do you use any method of family planning methods before (during) this pregnancy?

A .Yes

B.No

12-which family planning methods are you using before this pregnant? (Specify)…………………

13-who decides to use family planning in the household?

A. Husband/partner
B. Wife
C. Both

14- What are the reasons for not using family planning methods?

| | | |
|---|---|---|
| Infrequent sex/no sex | a. yes | b. no |
| Want to get birth | a. yes | b. no |
| Respondents opposed | a. yes | b. no |
| Husband/partner opposed | a. yes | b. no |
| Religion opposed | a. yes | b. no |
| Knows no method | a. yes | b. no |
| Knows no source (where to find) | a. yes | b. no |
| Fear of side effect | a. yes | b. no |
| Inconvenient to use | a. yes | b. no |
| No reason | a. yes | b. no |
| Others (specify) | a. yes | b. no |

16-Do you use/accept any family planning methods now (post abortion)?

A. Yes B. no

17-Have you ever been counseled by health professionals about family planning methods?

A. Yes B. no

18 -Do you think you have got enough information from the counseling?

A. Yes B. no

19 -Does your husband/partner know that you are using family planning method?

A. Yes B. no

**Part 4, Abortion related data**

20-Do you know about post abortion family planning?

A. Yes B. no

21. From where did you hear about post abortion family planning?

A. Mass media c. health professional
B. Neighbors/ friend d. other (specify)

22-Do you believe in the importance of post- abortion family planning?

A. Yes B. no

ቅጽ: 2 በአማረኛ የተተረጎ መቃለመጠይቅ

እኛ የአልካን ጤና ሳይንስ ኮሌጅ ማህበረሰብ አቀፍ ጤና አጠባበቅ ተማሪዎች ስንሆን የምናደርገዉም ጥናት ማሪ ስቶፕስ ኢንተርናሽናል ክሊኒክ እና በደሴ ጤና ጣቢያ ከዉርጃ በኋላ ስለሚወሰዱየቤተሰብ ምጣኔና ተያይዘዉ የሚመጡ ችግሮችን ለይቶማወቅ ነዉ።

ጥያቅዎችን ለመመለስ ፍቃደኛ ነዎት

**ክፍል 1.የማህበራዊ፣ ኢኮኖሚያዊናድሞግራፊያዊ ሁነቶች**

1. እድሜዎ ሀ. 15-19 ለ. 20-24 ሐ. 25-29 መ. ከ30 በላይ

2. ክልል ሀ. ኦሮሞ ለ. አማራ ሐ. ትግራይ መ. ጉራጌ ሠ. ሌላ

3. ሃይማኖት

ሀ. ኦረቶዶክስክርስቲያን ሐ. ካቶሊክ

ለ. ፕሮቴስታንት ክርስቲያን መ. ሙስሊም ሠ. ሌላ

4. አሁን የምትኖሪበት ቦታ

ሀ.ደሴ ለ. ዉጭሃገር ሐ.ክፍለሃገር

5. የጋብቻሁኔታ፡

ሀ. ያላገባች ለ. ያገባች ሐ. አግብታ የፈታች

መ. ባለቤቷ የሞተባት ሴት

6. ትምህርት ሁኔታ

ሀ. ያልተማረች ለ. መጻፍና ማንበብ የምትችል

ሐ. የመጀመሪያ ደረጃ ትምህርቷን ያጠናቀቀች

መ. የሁለተኛ ደረጃ ትምህቷን ያጠናቀቀች

ሠ. በኮሌጅ ተምራ የተመረቀች

7. ሥራ ሀ. የቤትእመቤት ለ. የመንግስት ተቀጣሪ

ሐ. ተማሪ መ. የቀን ሰራተኛ ሠ. ስራ የሌላት ረ. ሌላ

**ክፍል 2 ከወሊድ ጋር ተያይዞ የቀረቡጥያቄዎች**

8. ወልደሽ ታዉቂያለሽ ሀ. አዎ ለ. አላዉቅም

9. ከአሁንበፊትአስወርዶሽ ያዉቃል ሀ. አዎ ለ. አያዉቅም

**ክፍል 3 ከቤተሰብ ምጣኔ ጋር የተያያዙዳታወች**

10. የወሊድመከላከያ ዘዴ ተጠቅመው ያውቃሉ ሀ. አዎ ለ. አላዉቅም

11. መልሰዎ አዎ ከሆነየትኛኛዉን የወሊድመከላከያ ዘዴተጠቅመውያውቃሉ (ጥቀሽ)

12. በቤተሰቡ ዉስጥ የወሊድመከላከያ እንድጠቀሙ ዉሳኔ የሚሰጠዉ

ሀ. ባለቤትዎ ነዉ ወይስ ጓደኛዎ ሐ .ሁለታችሁም

ለ. እርሰዎ

13. የቤተሰብ ምጣኔ ያለማንም ጣልቃገብነት ይወስዱነበር

ሀ. አዎ ለ. አልወስድም

14. የቤተሰብ ምጣኔ ላለመጠቀም ምክናያትዎ ምን ነበር

- ✓ ጾታዊ ግንኙነት ስለማታደርጉና አልፎአልፎስለሆነ፡-

ሀ. አዎ ለ. አይደለም

- ✓ መዉለድስለምትፈልጉ፡

ሀ. አዎ ለ. አይደለም

- ✓ መዉለድስለማትፈልጉ፡-

ሀ. አዎ ለ. አይደለም

- ✓ ባልተቤትዎ ወይም ጓደኛዎ ስለሚቃዎሙ፡-

ሀ. አዎ ለ. አይደለም

- ✓ ሀይማኖትዎ ስለማይፈቅድ፡-

ሀ. አዎ ለ. አይደለም

- ✓ የመከላከያዘዴያለማዎቅ ሀ. አዎ ለ. አይደለም

- ✓ የትእንደሚገኝያለማዎቅ፡- ሀ. አዎ ለ. አይደለም

- ✓ ተጓዳኝ ችግሮችን በመፍራት፡-

ሀ. አዎ ለ. አይደለም

- ✓ ለአጠቃቀም ምቹአለመሆን፡- ሀ. አዎ ለ. የለም
- ✓ ምንም ምክናያት የለሽም፡- ሀ. አዎ ለ. አለኝ ሐ. ሌላ ካለ

15. ከዚህ ዉርጃበኋላ የቤተሰብ ምጣኔ ለመዉሰድዝግጁነሽ፡-

ሀ. አዎ ለ. አይደለሁም

16. ሥለቤተሰብ ምጣኔ በጤና ባለሙያ ምክር አግኝተሻል

ሀ. አዎ ለ. አላገኘሁም

17. በምክሩበቂየሆነመረጃአግኝቻለሁብለሽ ታስቢያለሽ

ሀ. አዎ ለ. አላስብም

18.ባለቤትሽ የቤተሰብ ምጣኔ እንደምትወስጅ ያዉቃል

ሀ አዎ ለ. አያዉቅም

## ክፍል 4 ከዉርጃ ጋር የተያያዙጥያቄዎች

19. ከዉርጃበሁዋላ ስለሚወሰዱየቤተሰብ ምጣኔ ታዉቂያለሽ

ሀ. አዎ ለ. አላዉቅም

20. ከዉርጃበኋላ መወሰድ ያለበትንየቤተሰብ ምጣኔ ከየትሰማሽ

ሀ. ከመገናኛ በዙሃን ሐ. ከጎረቤትወይም ጓደኛ

ለ. ከጤና ባለሙያ መ. ከሌላ

21. ከዉርጃበኋላ የቤተሰብ ምጣኔ መዉሰድ ጥቅም አለዉ ብለሽ ታምኒያለሽ

ሀ. አዎ ለ. አላምንም
